# Use of Physical Restraint in Psychiatry: Attitudes of Healthcare Providers and Ethical Considerations

**DOI:** 10.1192/j.eurpsy.2025.1510

**Published:** 2025-08-26

**Authors:** N. Sghaier, W. Belaguide, N. Kalboussi, S. Halleb, H. Ben Garouia, J. Mannai

**Affiliations:** 1 Psychiatry Deprtement, Ibn Jazzar university, Kairouan, Tunisia

## Abstract

**Introduction:**

Physical restraint in psychiatry is a widely used practice intended to protect patients from harming themselves or others, guided by strict procedures and monitoring. Recent reports and legal updates aim to regulate its use more closely

**Objectives:**

This study assesses the extent of physical restraint use and explores healthcare workers’ perceptions and experiences regarding this practice, focusing on ethical issues.

**Methods:**

Between April and May 2024, we conducted a cross-sectional descriptive study involving healthcare staff from psychiatry departments across Tunisia, including hospitals in Sousse, Monastir, Kairouan, Mahdia, Sfax, and Tunis. Participants were surveyed using a literature-based questionnaire, and data were analyzed with SPSS21 software.

**Results:**

The study included 16 men (28%) and 43 women (72%), predominantly aged 20-30 years (72%), with most participants from Kairouan (52%). Sixty-four percent of staff viewed physical restraint as a therapeutic tool. Opinions on its impact on the therapeutic alliance and physical integrity were mixed, with 33% considering it dehumanizing. The most common emotions reported were fear (58%) and pity (39%), while anxiety was the least reported (9%). Coping strategies included rationalization (63%) and discussing experiences with colleagues. Sixty-six percent of staff reported encountering ethical dilemmas, with varying frequencies. Views on patient consent were divided, with 42% opposing seeking consent, and differing opinions on obtaining consent from patients with good insight or in relapse.

**Conclusions:**

The study reveals diverse and complex attitudes towards physical restraint in psychiatry. It underscores the need for continuous training, ethical reflection, and efforts to align practices with ethical standards to mitigate negative impacts on staff.

**Disclosure of Interest:**

None Declared

